# Objective Diagnosis

**Published:** 1885-05

**Authors:** 


### OBJECTIVE DIAGNOSIS.

At the last annual meeting of the *American Medical Association*, in the section on *Obstetrics and Diseases of Women*, Dr. S. C. Gordon, of Portland, Maine, read an interesting and valuable paper, entitled "*Reasons for and Results of Some Cases of Tait's Operation.*"

Dr. Gordon's paper was suggested by an incident which occurred at the last *International Medical Congress*, at Copenhagen. Sir Spencer Wells, Bart., in criticism of the communication by Koeberlé, of Strasbourg, said: "As regards castration of women for nervous disease, we might just as well go about the country cutting men for similar disease as to do what is being done in removing ovaries of women. I believe that in no case should the ovaries of a woman be removed, unless we can demonstrate beyond all doubt that there is actual disease." In reply to Dr. Gordon's question: "Do you mean by demonstration only that which can be made by touch?" Sir Spencer Wells said: "Yes, most emphatically!"

Dr. Gordon, in the paper to which allusion has just been made, after referring Sir Spencer Wells' adverse criticism of Tait's operation to his well-known personal aversion to that distinguished surgeon, cited seven cases in which he had performed Tait's operation, with benefit to his patients, in the absence of tangible evidence of ovarian or tubal disease. He thought that a critical analysis of subjective symptoms, through a sufficiently long period of time, in the utter absence of positive evidence elicited by touch, could establish a diagnosis of a pathological condition of the uterine *adnexa* with such a degree of probability as to indicate operative procedure.

The subjective symptoms, to which special importance was attached, are pre-menstrual pain, irregularity of menstrual function, circulatory disturbances, and a peculiar mahoganybronze color of the skin of the face. This peculiar *cutis ænea* resembles closely the bronze discoloration observed in Addison's disease.

Dr. Gordon's paper excited much discussion. Dr. William H. Wathen, of Louisville, Ky., thought the doctrine of operation, without distinct objective evidence, was erroneous, absolutely, and pernicious in its tendencies. We cannot but think Dr. Wathen's stricture well founded. All the rational signs and extraneous symptoms deserve to be carefully inquired into and noted, but the testimony derivable from them cannot, in the present state of our knowledge, amount to more than a high degree of *probable* evidence. It is only in very exceptional cases that such a degree of probability is established as would justify such a radical procedure as Tait's operation.

Moreover, the *tactus eruditus* is capable of wonderful development. The methods at our disposal for the local examination of the female genital tract are numerous, and in the majority of cases adequate.

By means of inspection of the vulva and abdomen, auscultation and percussion of the abdomen, palpation of the abdomen, digital examination, vaginal, rectal, vesical, conjoined examination, vagino-abdominal, recto-abdominal, vesico-abdominal, digital eversion of the rectum, every portion of so small a space as the pelvic cavity can usually be thoroughly explored, and that, too, without serious injury to the patient. As important adjuvants, we have posture and anæsthetics.

It is well to remember, in this connection, the teachings of Oppolzer and Hebra. Oppolzer's famous diagnosis of strangulated hernia by palpation, after the rejection of the case at the Surgical Clinics of the Vienna General Hospital, is familiar to all students of the history of medicine. The elder Hebra was accustomed to say: "Gentlemen, recollect your pathological anatomy and make an objective diagnosis."

